# Decreased haemodynamic response and decoupling of cortical gamma-band activity and tissue oxygen perfusion after striatal interleukin-1 injection

**DOI:** 10.1186/s12974-016-0664-x

**Published:** 2016-08-24

**Authors:** Natasha Bray, Fiona E. Burrows, Myles Jones, Jason Berwick, Stuart M. Allan, Ingo Schiessl

**Affiliations:** 1 Faculty of Life Sciences, The University of Manchester, Oxford Rd, Manchester, M13 9PT UK; 2 Department of Psychology, University of Sheffield, Western Bank, Sheffield, S10 2TP UK

**Keywords:** Interleukin-1β, Neurovascular coupling, Haemodynamics, Cortical oxygenation, Optical imaging spectroscopy

## Abstract

**Background:**

Neurovascular coupling describes the mechanism by which the energy and oxygen demand arising from neuronal activity is met by an increase in regional blood flow, known as the haemodynamic response. Interleukin 1 (IL-1) is a pro-inflammatory cytokine and an important mediator of neuronal injury, though mechanisms through which IL-1 exerts its effects in the brain are not fully understood. In this study, we set out to investigate if increased cerebral levels of IL-1 have a negative effect on the neurovascular coupling in the cortex in response to sensory stimulation.

**Methods:**

We used two approaches to measure the neuronal activity and haemodynamic changes in the anaesthetised rat barrel somatosensory cortex in response to mechanical whisker stimulation, before and for 6 h after intra-striatal injection of interleukin-1β or vehicle. First, we used two-dimensional optical imaging spectroscopy (2D-OIS) to measure the size of the functional haemodynamic response, indicated by changes of oxyhaemoglobin (HbO_2_) and total haemoglobin (HbT) concentration. In the same animals, immunostaining of immunoglobulin G and SJC-positive extravasated neutrophils was used to confirm the pro-inflammatory effects of interleukin-1β (IL-1β). Second, to examine the functional coupling between neuronal activity and the haemodynamic response, we used a ‘Clark-style’ electrode combined with a single sharp electrode to simultaneously record local tissue oxygenation (partial pressure oxygen, pO_2_) in layer IV/V of the stimulated barrel cortex and multi-unit activity (MUA) together with local field potentials (LFPs), respectively.

**Results:**

2D-OIS data revealed that the size of the haemodynamic response to mechanical whisker stimulation declined over the 6 h following IL-1β injection whereas the vehicle group remained stable, significant differences being seen after 5 h. Moreover, the size of the transient increases of neuronal LFP activity in response to whisker stimulation decreased after IL-1β injection, significant changes compared to vehicle being seen for gamma-band activity after 1 h and beta-band activity after 3 h. The amplitude of the functional pO_2_ response similarly decreased after 3 h post-IL-1β injection, whereas IL-1β had no significant effect on the peak of whisker-stimulation-induced MUA. The stimulation-evoked increases in gamma power and pO_2_ correlated significantly throughout the 6 h in the vehicle group, but such a correlation was not observed in the IL-1β-injected group.

**Conclusions:**

We conclude that intra-striatal IL-1β decouples cortical neuronal activity from its haemodynamic response. This finding may have implications for neurological conditions where IL-1β plays a part, especially those involving reductions in cerebral blood flow (such as stroke).

## Background

Neuroinflammation following a brain injury or stroke worsens the effects of the initial injury [1]. One of the major and most studied mediators of neuroinflammation is interleukin-1β (IL-1β). IL-1β does not damage the brain by itself [2–4], but it is heavily implicated as exacerbating cell death following a CNS injury such as stroke (for review, see [5]). In the context of stroke, diagnosis and treatment must be carried out within hours of ischaemic onset. The administration of IL-1 antagonists has been shown to reduce neuronal loss following stroke [6], traumatic brain injury (TBI) [7] or seizure activity [8] in various animal models. However, the mechanisms underlying the deleterious effects of IL-1β in acute brain injury are not yet well understood but are thought to be numerous and complex.

Several studies have documented the acute pro-inflammatory effects of IL-1β on neutrophil infiltration and blood-brain barrier (BBB) permeability [2, 9, 10], but few have investigated the effects of IL-1β on the functionally relevant increases in neuronal activity in response to sensory stimulation and the associated haemodynamic response. One study that did investigate IL-1β effects on cerebral blood flow (CBF) reported a temporary decrease in apparent diffusion coefficient 4–6 h following injection of IL-1β into the striatum [11]. The same study also found a temporary increase in striatal blood volume but did not examine the effects on functional haemodynamic responses or neuronal activity in the cortex in response to stimuli.

In this study, we investigate the acute effects of IL-1β on the haemodynamic response, that is, the transient, local increase of oxygenated blood supply in response to increased neuronal activity [12]. To do this, we used two-dimensional optical imaging spectroscopy (2D-OIS) to record the changes in the concentration of oxyhaemoglobin (HbO_2_) and total haemoglobin (HbT) within the well-characterised rat barrel cortex in response to mechanical whisker stimulation [13], before and after unilateral striatal injection of IL-1β. These recordings demonstrate that IL-1β induces a decrease in the haemodynamic response in the cortex, just hours after striatal injection. Moreover, we examined extravasated neutrophils and immunoglobulin G (IgG; an indicator of BBB permeability) as histological markers of neuroinflammation in the same animals and found an IL-1β-dependent increase in both of these markers in the ipsilateral brain hemisphere.

To investigate if those changes in the haemodynamic response after intra-striatal IL-1β injection are driven by decreased neuronal activity in the cortex, we used an action potential-oxygen (APOX) electrode, which allows the simultaneous recording of electric neuronal activity and tissue oxygenation. In this way, we were able to investigate how the changes in functional HbO_2_ response might translate into a decline in tissue oxygenation and whether a change in oxygenation might precede or follow any changes in neuronal activity. Specifically, we examined the local field potential (LFP) and multi-unit activity (MUA) in the putative layer IV/V of the barrel cortex (600 μm below the cortical surface), where mechanosensory inputs from the whisker pathway enter the somatosensory cortex [14].

LFP activity is associated with neuronal input arriving into the barrel cortex [15] and is correlated with a large demand for energy and corresponding haemodynamic response [16]. By contrast, MUA requires less energy and represents the ‘output’ of the cortical layer that is directed towards other processing regions [17]. As the blood-oxygen-level-dependent (BOLD) signal observed using fMRI results from the ‘washing away’ of deoxygenated blood by the influx of fresh oxygenated blood, it is thus commensurate to the changes in the HbO_2_ concentrations recorded with our optical imaging spectroscopy. The dissociation between LFPs and MUA allows for the distinction between energy demand and neural activity. MUA is reduced if cells are damaged or dead but can be maintained even when there is a disruption in blood flow, whereas normal LFP activity reflects efficient haemodynamic coupling as well as neuronal activity [15].

Two of the commonly used electroencephalogram (EEG) frequency bands within the LFP range that are most associated with the BOLD signal and thus haemodynamics are the gamma- and beta-frequency bands (40–100 and 13–24 Hz, respectively) [18]. There is a well-documented correlation between the size of a stimulus-evoked increase in gamma- and/or beta-band activity and the size of the localised response in blood flow to the active cortex [17, 19, 20]. The established association between these forms of neural activity and compensatory blood flow therefore provides a neurovascular coupling relationship that may be perturbed by brain injury.

Thus here, we examine the acute effects of IL-1β on neural activity and cortical haemodynamics in response to mechanical stimulation of the barrel cortex to investigate whether IL-1β exacerbates stroke and other brain injuries by negatively impacting upon neurovascular coupling.

## Methods

### Animals

All experimental procedures using animals were conducted in accordance with the UK Animals (Scientific Procedures) Act, 1986, and approved by the Home Office and the local Animal Ethical Review Group, University of Manchester, UK. Male Lister hooded rats (Charles River, UK) weighing 250–400 g were housed in cages of up to five, at 21–23 °C and 60 ± 5 % humidity, with a 12-h light-dark cycle and with access to standard rat chow and water ad libitum.

Each of the two groups (vehicle and IL-1β) for the 2D-OIS study and immunohistochemistry contained five animals. For the APOX probe experiments—in which MUA, LFPs and partial pressure oxygen (pO_2_) responses were recorded—animals were only included in each analysis if they exhibited a reliable response with regard to that parameter on three consecutive whisker stimulation experiments prior to injection. As such, these analyses comprised differing *n* numbers: for MUA analysis, vehicle *n* = 4 and IL-1β *n* = 6; for LFPs, vehicle *n* = 4 and IL-1β *n* = 5; and for pO_2_, there was an *n* = 5 in both groups. The correlation of pO_2_ responses and LFP responses (in the form of gamma or beta oscillation power) thus included paired responses from four animals per group. Data from this study can be made available on request after publication and intellectual property agreement.

### Surgical preparation

Anaesthesia was induced with a 4 mL/kg intraperitoneal injection of 25 % Hypnorm (0.315 mg/mL fentanyl, 10 mg/mL fluanisone; Roche, UK) and 25 % Hypnovel (1 mg/mL midazolam; VetaPharma Ltd., UK) in sterile water (Braun, UK). Once the pedal reflex was lost, a tracheotomy was performed.

After the animals were transferred to a stereotaxic frame (Kopf Instruments, USA), they were artificially ventilated with 1 % isoflurane (Abbott, Berkshire, UK) in room air using a Zoovent Jetsys ventilator (Universal Lung Ventilators Ltd., UK). End-tidal CO_2_ was monitored using a Capnogard ETCO_2_ (Novametrix Medical Systems Inc., USA) and kept between 25 and 35 mHg for the duration of the experiment to ensure adequate ventilation and to prevent hypercapnia, which may affect baseline perfusion as well as the haemodynamic response [21]. The animal’s core temperature was monitored using a rectal probe and maintained at no less than 37 °C by a homeothermic heat mat (Harvard Apparatus, UK). A Tiger Pulse Veterinary Oximeter (Thames Medical) with a rectal probe was used to measure peripheral capillary oxygen saturation (SpO_2_). Electrocardiogram (ECG), SpO_2_ and body temperature were recorded at regular intervals throughout the experiment.

After exposing the skull, a hole in the cranium for the intra-striatal injection was drilled using a dental drill with a 0.8-mm drill bit (Messinger, Germany) at stereotaxic co-ordinates 2.7 mm ipsilateral and 0.5 mm anterior to the bregma [22]. For electrode recording experiments, a shallow dimple was drilled in the cranium at 4 mm contralateral and 4 mm posterior relative to the bregma, into which a flat-point screw was inserted to act as a ground electrode.

For OIS, a rectangular area of bone measuring ~4 × 4 mm over the somatosensory cortex was thinned slowly and uniformly to translucency (100 μm) using a dental drill, intermittently cooled using chilled (4 °C) 0.9 % saline (Braun, UK) to prevent heat damage. Any bleeding vessels in the cranium were sealed with bone wax (Ethicon, UK), and a well was made around the imaging area using dental cement (Kemdent, UK). The well was filled with warmed (37 °C) 0.9 % saline and sealed with a 1.2-mm-diameter glass coverslip (Scientific Laboratory Supplies Ltd., UK) using Vaseline (Unilever, UK) to prevent evaporation.

### Optical imaging spectroscopy

OIS was used to measure the functional haemodynamic response to whisker stimulation before and every 20 min after striatal injection of IL-1β or vehicle (see the ‘Striatal injection’ section below).

A charge-coupled device (CCD) 1M30 Pantera camera (Teledyne DALSA, Canada) was positioned normal to the imaged surface, which was illuminated with light from a halogen projection lamp (6958 24 V 250 W G6.35, Phillips, UK) that passed through one of three alternating wavelength filters (550 ± 10 nm, 560 ± 10 nm, 577 ± 10 nm) transmitted via a liquid light guide. The field of view was focused on the cortical surface vessels and centred on the barrel cortex.

Filters were alternated by a Lambda DG-4 filter changer (Sutter Instruments, USA) in synchronisation with the camera to obtain alternating frames for each wavelength, with an effective frame rate of 8 Hz per wavelength. The light source was powered by an uninterrupted CPX200 PowerFlex power supply (TTi) to allow stable illumination. Image acquisition, stimulus generation and filter alternation were synchronised and mediated using a BNC 2090 (National Instruments, UK) connected to a PCI data acquisition card (PCI-MIO-16E-4, National Instruments, UK), all controlled by MATLAB (MathWorks Inc., USA).

### Whisker stimulus

Mechanical stimulation of the E1 whisker was achieved using a piezoelectric ceramic actuator (Physik Instrumente GmbH & Co. KG, Germany) with an 8-Hz stimulus driven by an alternating current amplifier (E-650.00 LVPZT; Physik Instrumente GmbH & Co. KG, Germany). One imaging experimental stimulus presentation session consisted of 30 trials, each 13 s long, comprising a 1-s pre-stimulus, 4-s stimulation, followed by 8-s recovery. After the ‘baseline’ imaging (that is, that conducted prior to injection) and subsequent injection of vehicle or IL-1β, imaging experiments or electrode recordings with stimulus presentation were carried out every 20 min up to 6 h after injection.

### Striatal injection

After baseline OIS or electrode recordings, animals received direct striatal injection of a 1-μL solution of either vehicle or 50 ng/μL human recombinant IL-1β (hrIL-1-β, hereafter referred to as IL-1β; National Institute for Biological Standards and Control (NIBSC), UK) in vehicle. Solutions also contained a small amount of mistral blue in order to visualise the solution during injection and to confirm the site of injection after the experiment. All solutions were made using the vehicle: 0.5 % sterile endotoxin-free bovine serum albumin (BSA; Sigma-Aldrich, UK) in phosphate buffer solution (PBS). All injected solutions were mixed on the day of injection from stock aliquots that were stored at −80 °C and were kept on ice until use.

Injections were made with a pulled glass microneedle (Sigma-Aldrich, UK) attached to a 5-mL syringe. Infusions were made at an approximate rate of 0.5 μL/min. The injection site was 0.7 mm anterior, 2.7 mm lateral to the bregma, and 5.5 mm ventral to the dura, which was opened with a 24-gauge needle just before injection to allow easy penetration of the microneedle. After injection, the microneedle was kept in place for 5 min before removal to prevent reflux of the solution.

### Immunohistochemistry

At the end of each experiment, each animal was euthanised with an i.p. injection of 2 mL sodium pentobarbitone (Pentoject, Animalcare Ltd., York, UK) and perfused transcardially with 4 °C 0.9 % saline nitrate and then 4 % paraformaldehyde for 10 min. The brains were removed, fixed overnight in 4 % paraformaldehyde and post-fixed in 30 % sucrose/PBS for >24 h. Coronal slices with 30-μm thickness were cut in series 360 μm apart on a sledge microtome and stored at −20 °C in cryoprotectant (6.6 g/L disodium hydrogen orthophosphate and 0.79 g/L sodium dihydrogen orthophosphate in 30 % ethylene glycol and 20 % glycerol in distilled H_2_O) until needed for immunohistochemical analysis.

Immunostaining of immunoglobulin G (IgG) and neutrophils was performed on free-floating sections. To identify neutrophils, sections were washed for 10 min three times with PBS, incubated with 0.3 % hydrogen peroxide (Sigma) for 10 min and washed in PBS, again for 10 min three times. The sections were then incubated in 2 % normal goat serum (Vector Labs, Peterborough, UK) in primary diluent (0.3 % Triton in PBS) for 1 h before being incubated overnight at 4 °C with the primary anti-SJC antibody (raised in rabbit) at a dilution of 1:300 in primary diluent. The next day, the slices were washed three times with PBS and then incubated with a biotinylated anti-rabbit secondary antibody (1:500; Vector Labs) in primary diluent for 2 h. An ABC kit (Vectastain; Vector Labs) mix of components A and B in PBS (17.5 μL + 17.5 μL in 13 mL, respectively) was prepared 30 min before use. After washing in PBS as before, slices were incubated for 1 h in the ABC mix, before developing using 0.01 % diaminobenzidine (DAB; Sigma) and 0.005 % hydrogen peroxide in distilled water (dH_2_O). Samples were finally washed in PBS before mounting onto slides and coverslipping using DPX mounting medium (Sigma-Aldrich). For IgG immunostaining, the same steps were carried out as with the SJC (Sandra Jane Campbell), except no primary antibody step was required and a biotinylated anti-rat IgG antibody (made in goat; Vector Labs) diluted 1:500 in primary diluent was used instead of the secondary antibody.

No information regarding group assignation was present on the slides; thus, experimenters were ‘blind’ to this information until after analysis had been carried out, to prevent experimenter bias.

To assess breakdown of the BBB as reflected by IgG extravasation, the ratio between the mean pixel values of the contralateral and ipsilateral hemispheres of each section immunostained with IgG was found by selecting a region of interest around each hemisphere on a photomicrograph and measuring the intensity of each region using ImageJ software (US National Institute of Health). Since DAB staining is not stoichiometric [23] and so cannot be compared between animals, this method was preferred because the contralateral hemisphere served as a control for individual animals. Any effect of oedema on the level of IgG immunostaining was diminished by averaging over the area of the whole hemisphere in each section.

The number of SJC-positive neutrophils in 0.56 × 0.42 mm areas was also obtained by counting manually from photomicrographs of the cortex and striatum of three sections per brain around the injection site at ×10 magnification using ImageJ.

### Electrophysiological recordings

The APOX electrode (Unisense, Aarhus, Denmark) allows for simultaneous recording of local tissue oxygenation and electrical field potentials from the same volume of brain tissue. Before insertion into the cortex, the electrode was switched on, left to stabilise for >30 min and calibrated as per the manufacturer’s instructions before each insertion.

After calibration, a small craniotomy was performed over the E1 barrel as established by optical imaging and the dura cut to allow the fragile probe to enter the cortex. The APOX probe was lowered normal to the surface of the active barrel cortex 600 μm into layer IV/V, at a rate of approximately 5 μm/s, using a custom-built micro drive (uD-800B Micro Drive Controller). The position of the probe was then further adjusted on a micrometre scale to find a consistent neural response to manual mechanical stimulation of the E1 whisker. As a control, the ipsilateral whiskers were stroked manually to ensure that the signal only came from contralateral whisker movement. The electrode was shielded from electrical noise from the piezoelectric actuator. The probe’s tissue oxygenation electrode was grounded on the head stage and connected to a two-channel PA-2000 picoammeter (Unisense, Denmark), which displayed the tissue oxygenation while the signal was also transferred to the I/O board.

The broadband electrical signal from the APOX neural electrode was filtered for 50 Hz using a HumBug 50/60 Hz Noise Eliminator (Quest Scientific, Vancouver, Canada) and then split into two channels for high-pass and low-pass filtering with separate amplification. The first contained the lower frequencies (5–300 Hz) associated with LFP activity. The other channel carried higher frequencies associated with MUA (300–3000 Hz). The tissue oxygenation data from the PA-2000 was recorded via a third channel of the National Instruments I/O board. All channels were recorded at a sampling rate of 20 kHz.

### OIS analysis

Automated feature extraction of the images that were recorded within the region of interest (ROI) was applied where possible to prevent experimenter bias. Image frames obtained from OIS experiments were grouped according to wavelength—namely 550, 560 and 577 nm. These single-wavelength image stacks were then averaged over the 30 stimulus trials. After averaging, the data was processed using a modified Beer-Lambert equation which utilises path-length scaling algorithm (PLSA) [13] to estimate changes in HbT and HbO_2_ producing three-dimensional cortical maps for those estimates comprised of the two-dimensional cortical maps over time. Within those three-dimensional data stacks, the area responding to the mechanical whisker stimulation, i.e. the barrel location, was extracted by automatically selecting pixels that had a larger than 1.5 standard deviation (SD) change between the pre-stimulus and stimulus periods and then excluding large cortical vessels. These pixels were then averaged within each recorded frame to give the one-dimensional time courses of the HbT and HbO_2_ components. From these time series, the peak response to stimulation relative to the pre-stimulus level of the HbT and HbO_2_ time series was found for each imaging experiment then averaged for each animal. To allow comparison between animals, responses after the injection were normalised to the response before injection 100 %.

### Analysis of pO_2_ responses

First, the picoampere reading from the APOX electrode PA-2000 was transformed into μmol/L concentrations of oxygen by linear regression between the calibration data points and then averaged across the 30 trials. For each experiment, the pre-stimulation value was calculated by averaging the first second of the trial-averaged data. To extract the post-stimulus pO_2_ value in response to the 4-s mechanical whisker stimulation, we averaged 1 s worth of data at the end of the stimulation (specifically between 4 and 5 s). The post-stimulus value was then expressed as a percentage of the pre-stimulus measurement to express the stimulus-evoked change ΔpO_2_, as previously reported in studies using the APOX probe [24]. In order to compare between the vehicle and IL-1β groups on how ΔpO_2_ varied in the 6 h following injection, we normalised the pre-injection value of ΔpO_2_ for each animal to 0 by subtracting the pre-injection value from all subsequent ΔpO_2_ values.

Animals were excluded from the analysis if tissue oxygenation recordings prior to intra-striatal injection did not display the typical expected canonical response function, as has been reported previously in the literature ([24]; namely a small initial dip and subsequent overshoot starting before the end of the stimulus). After excluding subjects on this basis, there remained five animals in the pO_2_ analysis for each condition (vehicle and IL-1β infusions).

### Analysis of MUA

**Fig. 1:**
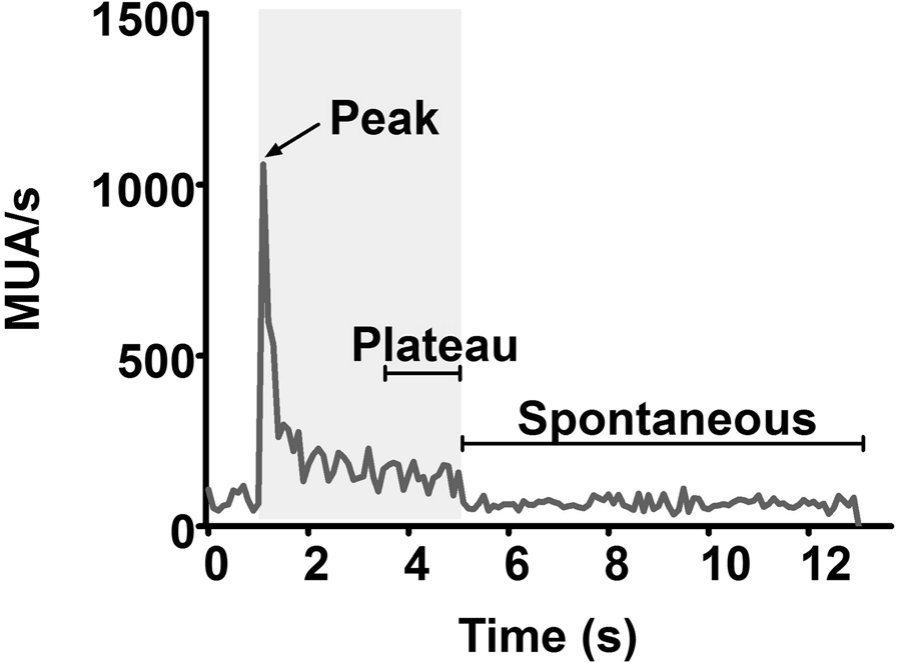
Example MUA spike density histogram indicating the analysed MUA parameters. At the stimulus onset (1 s; stimulus represented by *grey shading*), there is a peak in MUA. MUA then typically rapidly reduces to a ‘plateau’ level. The peak was isolated and quantified in this study by averaging activity in the last 1.5 s of the stimulus, adding this to the average ‘spontaneous’ activity and taking these away from the peak value

MUA was analysed by thresholding the electrophysiological data that had been filtered between 300 and 3000 Hz at 3 SD of the recording. A matrix was created to represent the incidence of the resulting ‘spikes’ in time within each of the 30 trials of each experimental session. In each trial, habituation to the stimulus occurred after 100–400 ms post-stimulus presentation in all animals. Trial-averaged spike rates within 100-ms time bins were calculated. The value of the maximum spike rate at the onset of the stimulus minus the average spike rate within the last 1.5 s (16 x 100 ms bins) of the stimulus period was normalised to the average spike rate after stimulus offset, or ‘(Peak − Plateau)’/‘Spontaneous’ as indicated in the Fig. 1. The calculated values of the peak spike rates were normalised to the pre-injection values for comparison of recordings before and after striatal injection.

### Analysis of LFP activity

**Fig. 2:**
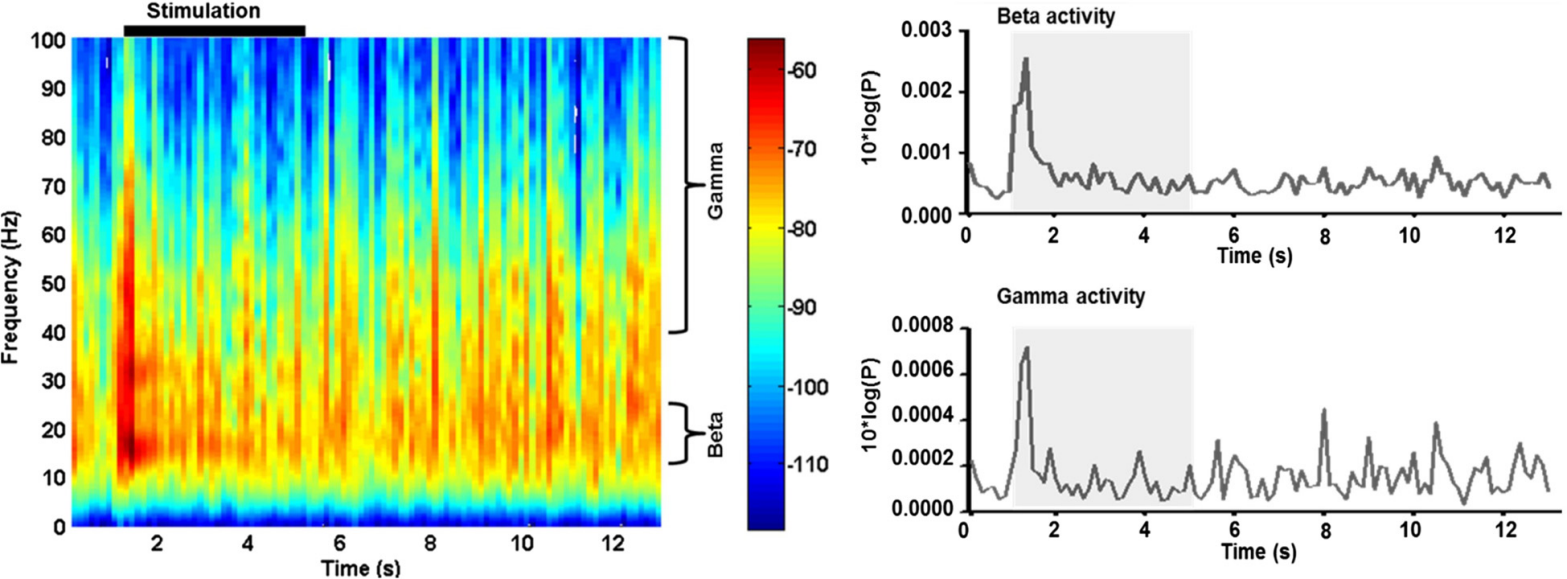
Analysis of gamma- and beta-frequency band LFP activity. (*Left*) Example 10*log(power) spectrogram (with window width 0.5 s, 0.25-s overlap and in 0.2-Hz intervals) showing averaged over 30 trials with 1-s pre-stimulus, 4-s whisker stimulation (indicated by the *black bar*) followed by 8-s recovery. Gamma (40–100 Hz) and beta (13–24 Hz) frequency bands are indicated. Note the marked increase in all frequencies at the onset of the stimulus at 1 s. (*Right*) Gamma- and beta-frequency bands averaged to show activity through the average trial. Again, note the increase in activity at the onset of the stimulation (*grey box*), which reduces to pre-stimulus levels by ~1 s into the stimulus

Spectrograms were calculated in MATLAB using 200-ms time windows with 100-ms overlap. Gamma (40–100 Hz) and beta (13–24 Hz) frequency bands were found to best reflect the pO_2_ response, as previously reported [18]. Therefore, these frequency bands were selected for further analysis. A mean spectrogram of the LFP recordings was calculated by averaging the spectrograms of the 30 trials per experimental session (Fig. 2 left). From these mean spectrograms, beta- and gamma-band activity was extracted by averaging across the respective frequency range (Fig. 2 right). The stimulus-evoked change in the amplitude of the gamma and the beta power was calculated by subtracting the peak response at the onset of the stimulus (at 1 s), minus the average gamma and beta power in the last 2.5 s of the 4-s stimulus. As with the previous data, the amplitudes of the stimulus-evoked LFP responses over the 6 h after injection were normalised to the pre-injection value.

### Statistical analyses

Statistical analyses were carried out using MATLAB or GraphPad (Prism). OIS analysis of the HbT and HbO_2_ responses, MUA, LFPs and pO_2_ was compared over time and between groups using repeated-measure analysis of variance (ANOVA) with Sidak post hoc tests. In this analysis, the pre-injection values were excluded, as, after normalisation to this time point, they have zero variance. Correlations between parameters were quantified using Pearson’s *r* rank coefficient. In order to assess if the injection of vehicle or IL-1β caused a significant change to the pre-injection measurements over time within a group, we applied a repeated-measure one-way ANOVA with Bonferroni correction for multiple comparisons.

Counts of SJC-positive neutrophils were analysed with two-way ANOVA using a Sidak post hoc test. Measures of IgG immunostaining were compared using unpaired Student’s *t* tests. Statistical significance was taken at the 5 % level.

## Results

### Striatal injection of IL-1β reduces the haemodynamic response to sensory stimulation

**Fig. 3:**
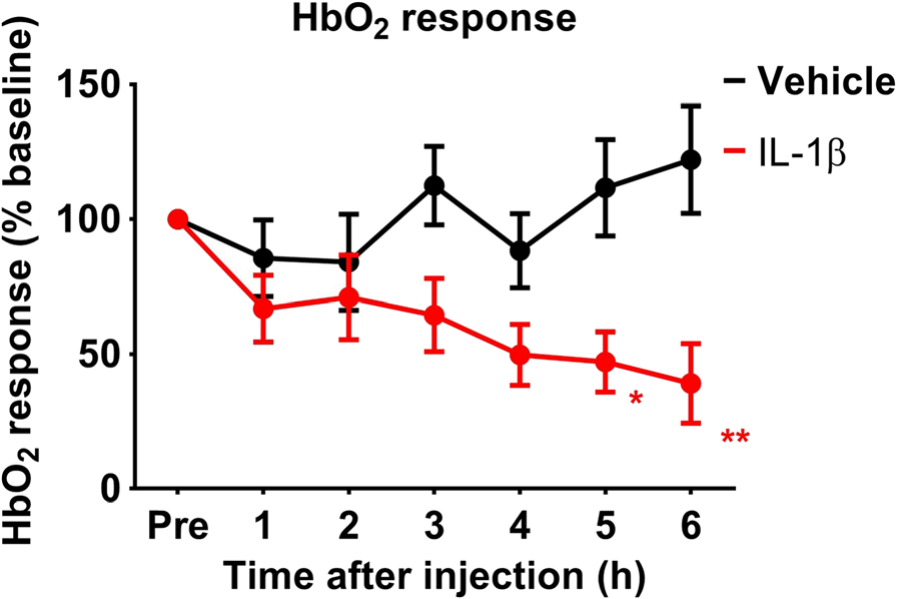
Stimulus-evoked HbO_2_ responses. Graph showing the change in the normalised amplitude of the HbO_2_ response before (*Pre*) and up to 6 h after striatal injection of vehicle or IL-1β (*n* = 5 each; data shown as mean ± SEM); * *p* < 0.05; ** *p* < 0.01, according to repeated-measure ANOVA excluding the pre-injection data points, with Sidak post-test

In this experiment, we used the size of the normalised stimulus-evoked change in the HbO_2_ and HbT as a measure of the change in the haemodynamic response. After baseline recording, we injected the ipsilateral striatum with IL-1β (*n* = 5) or vehicle (*n* = 5) and recorded the haemodynamic response to whisker stimulation following injection for 6 h. Mean baseline HbO_2_ responses before normalisation were not significantly different between groups prior to injection (*t* = 0.877, *p* = 0.406) showing the stability of the preparation. Animals injected with IL-1β showed a decline in their HbO_2_ responses to whisker stimulation compared to the vehicle group (*F*(1) = 7.373, *p* = 0.026) (Fig. 3). The difference between the two groups reached significance at 5 h post-injection and lasted until the end of the experiment (6 h).

When looking at the change within the group, striatal injection of vehicle did not induce a significant decline in cortical HbO_2_ responses (*F*(6) = 1.217, *p* = 0.332). By contrast, HbO_2_ responses were significantly decreased in animals after the injection of IL-1β (*F*(6) = 5.448, *p* = 0.005)—an effect that became apparent at 4 h post-injection.

**Fig. 4:**
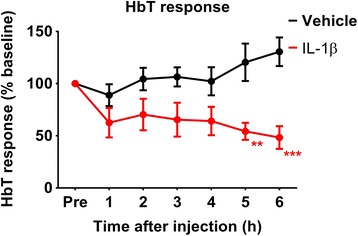
Stimulus-evoked HbT responses. Amplitudes of the HbT response to whisker stimulation pre- and up to 6 h post-injection of vehicle or IL-1β. *Points* represent the average of groups where two to four imaging experiments (each consisting of 30 trials) have been binned per animal. Data shown as mean ± SEM; * *p* < 0.05; *** *p* < 0.001, according to repeated-measure ANOVA excluding the pre-injection data points, with Sidak post-test

When comparing the change of the stimulus-evoked HbT responses between both groups over time, the response was significantly affected by the striatal injection of IL-1β (*F*(1) = 10.09, *p* = 0.0131) and exhibited an interaction effect (*F*(5) = 3.336, *p* = 0.013). Both groups were significantly different after 5 h until the end of the experiment (Fig. 4). When looking at the change of the HbT response within each group alone, there was a significant decrease after injection of IL-1β (*F*(6) = 4.811, *p* = 0.002) but not following the injection of vehicle (*F*(6) = 1.483, *p* = 0.217) again showing that the vehicle group remains stable.

These results show that the injection of IL-1β into the striatum induces a reduction in the size of the haemodynamic response to sensory stimulation compared to the vehicle group.

### Striatal injection of IL-1β is associated with extravasation of neutrophils and BBB breakdown

**Fig. 5:**
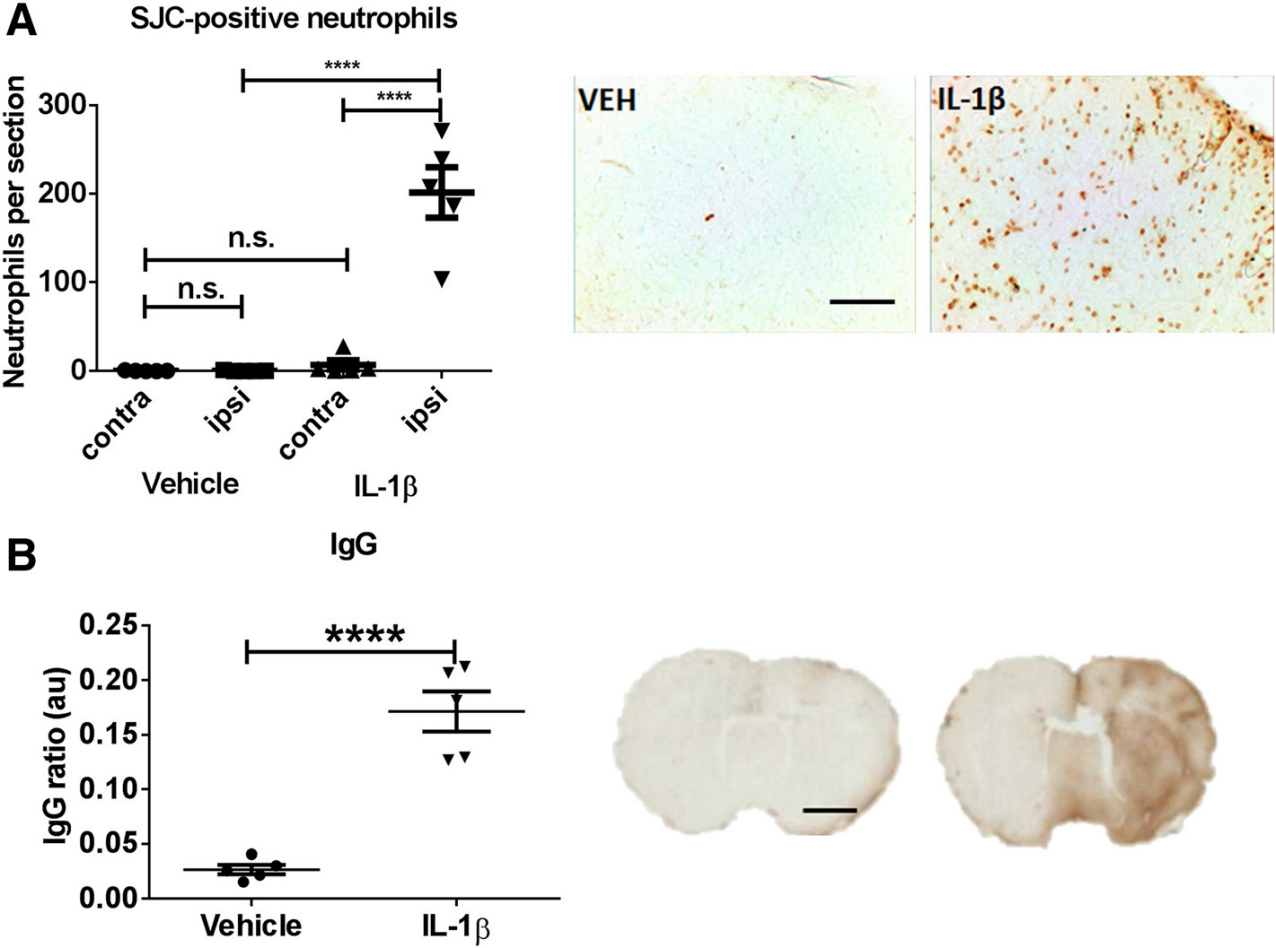
Cortical neutrophil recruitment and BBB breakdown. **a** Significant increase of neutrophils in the cortex of the injected hemisphere of animals that received intra-striatal injections of IL-1β- compared to vehicle-injected controls (**** *p* < 0.0001 comparing ipsilateral cortices in vehicle- versus IL-1β-injected animals and **** *p* < 0.0001 comparing ipsilateral versus contralateral cortex in IL-1β-injected animals using a two-way ANOVA with Sidak post-test). *Right*: representative photomicrographs of the ipsilateral cortex; *scale bar* 100 μm. **b** Increased IgG immunostaining (indicative of blood-brain barrier breakdown) was observed in the ipsilateral cortex of animals that received intra-striatal injections of IL-1β- than in vehicle-injected controls (**** *p* < 0.0001). *Right*: representative photomicrographs of IgG-immunostained brain sections from rats injected with vehicle (*left*) and IL-1β (*right*); *scale bar* 2.5 mm

To map the spread of inflammation after intra-striatal injection of IL-β or vehicle in both the ipsilateral and contralateral cortex, we used immunohistochemistry to measure the level of BBB breakdown and immune cell extravasation. More SJC-positive neutrophils were detected in the cortex of the IL-1β-treated animals than in vehicle-injected animals (*t* = 9.521, *p* < 0.0001). Moreover, the extravasation of neutrophils from the blood circulation was specific to the injected hemisphere, as there were significantly more of these cells in the ipsilateral cortex than in the contralateral hemisphere (*t* = 9.848, *p* < 0.0001) (Fig. 5a).

Similarly, there was increased BBB breakdown (as depicted by IgG extravasation) in the ipsilateral hemisphere of IL-1β-injected animals than in the ipsilateral cortex of vehicle-treated animals (*t* = 7.652, *p* < 0.0001) (Fig. 5b). These results confirm previous findings [3] that intra-striatally injected IL1β induces inflammatory effects throughout the injected hemisphere, including the ipsilateral cortex.

### Striatal injection of IL-1β leads to a decline of stimulus-associated LFP activity

In a separate cohort of animals (vehicle *n* = 4, IL-1β *n* = 5), we measured the stimulus-evoked changes in gamma- and beta-band activity before and after intra-striatal injection of IL-1β or vehicle using the APOX electrode. Across both groups, there was a significant effect of time on the amount of gamma activity induced by mechanical whisker stimulation after injection (*F*(5) = 3.769, *p* = 0.008). The difference between both treatment groups reached significance after 6 h (Bonferroni post-tests; *t* = 3.301, *p* < 0.05).

**Fig. 6:**
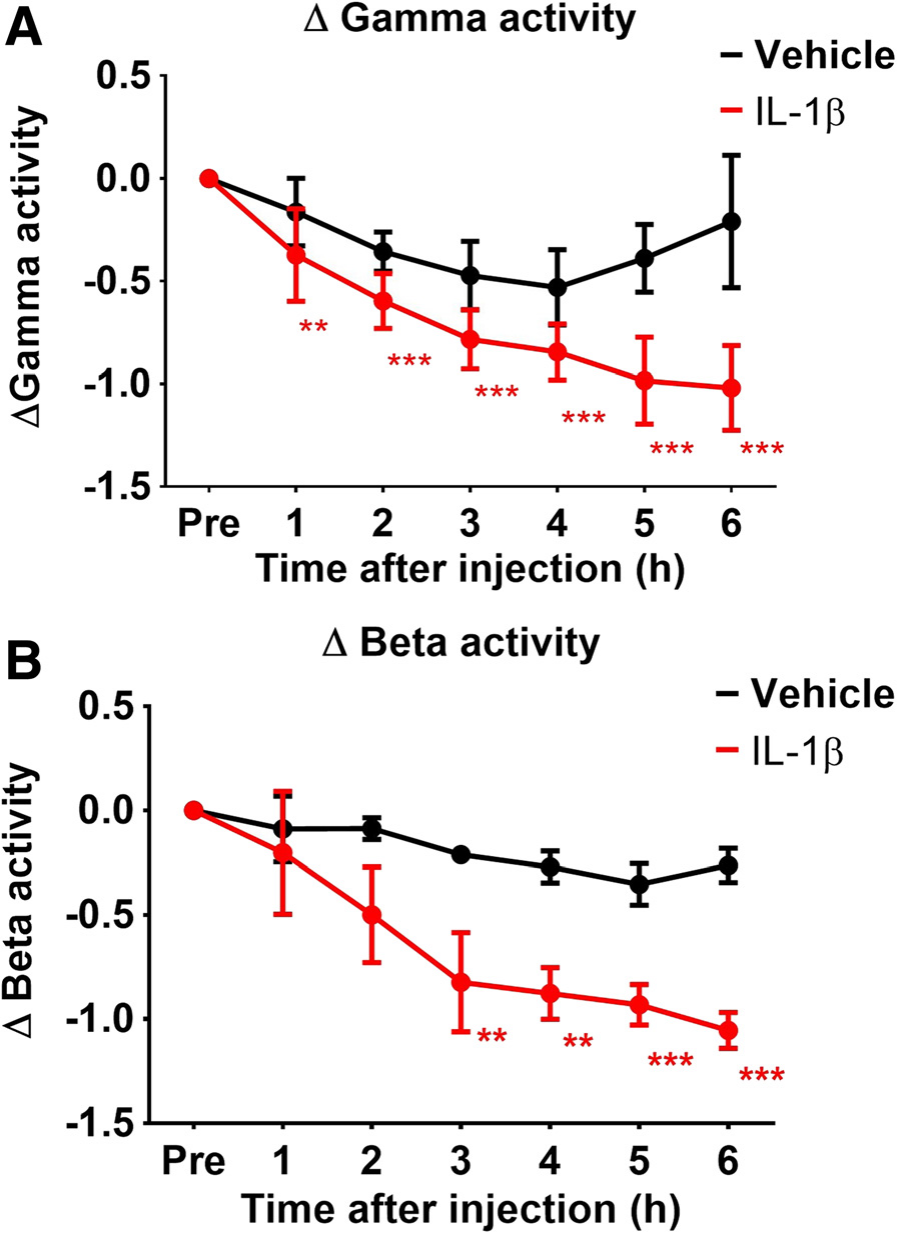
Change in gamma- and beta-band power peak amplitudes. Graphs showing the averaged changes in the log amplitude of the **a** gamma-band frequency and **b** beta-band frequency activity peaks at the onset of the stimulus in groups before and after striatal injection. The *asterix* show the significance of the change within each group compared to the pre-injection values. Mean ± SEM; ** *p* < 0.01; *** *p* < 0.001; repeated-measure one-way ANOVA before normalisation to pre-injection time point with Bonferroni post-tests versus *Pre*

Within-group analyses (using non-normalised data) found that whereas vehicle-treated animals suffered no significant loss in gamma responses (*F*(27) = 1.501, *p* = 2.338) over the full 6 h, gamma activity in IL-1β-treated animals was quickly and markedly diminished (*F*(27) = 14.22, *p* < 0.0001) just 3 h after injection until the end of the experiment at 6 h (Fig. 6a).

There was an effect of time (*F*(5) = 7.103, *p* = 0.0001) and of IL-1β treatment (*F*(1) = 7.604, *p* = 0.028) on beta activity responses across the groups. Similarly to the gamma responses, IL-1β-injected animals exhibited significantly less beta activity at 6 h after injection compared to the vehicle (*t* = 3.359, *p* < 0.05). Within-group analyses found that whereas the vehicle-treated animals showed no decline in beta activity (*F* = 1.960, *p* = 0.144), injection of IL-1β led to significantly lower levels of beta activity by 3 h after injection (*F* = 6.986, *p* < 0.0001) (Fig. 6b). The data therefore demonstrates that the striatal injection of IL-1β negatively affected these cortical oscillations, as early as 3 h after injection.

### Cortical pO_2_ responses are reduced after striatal injection of IL-1β

Considering that IL-1β injection leads to a reduction in stimulus-evoked HbO_2_ and HbT haemodynamic responses as well as changes in LFP activity compared to the vehicle group, we investigated how these observations translate into changes in cortical pO_2_, recorded simultaneously in the same animals as the LFP and MUA data.

**Fig. 7:**
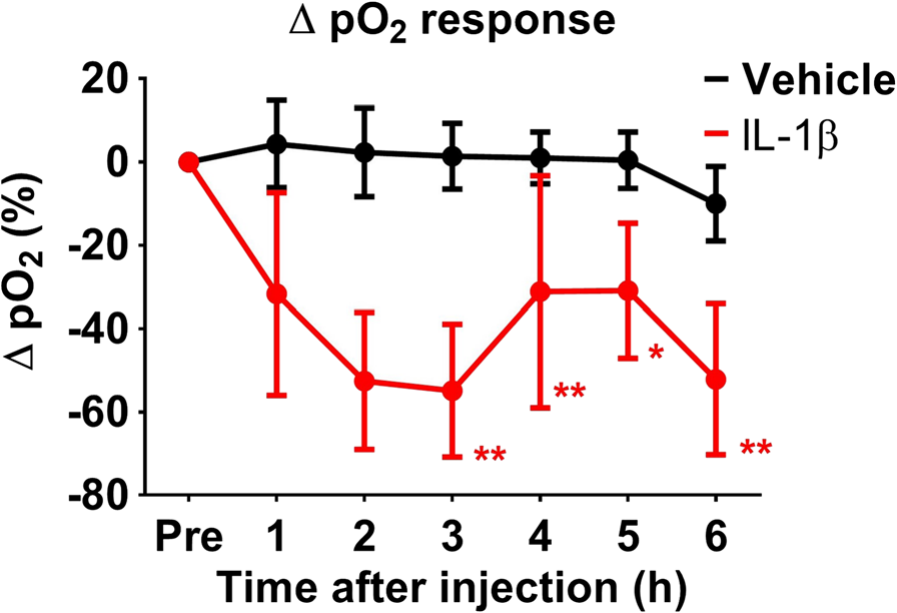
Stimulus-evoked cortical pO_2_ responses. The change in the size of the oxygenation response (% baseline concentration) to whisker stimulation is shown. Animals injected with IL-1β showed pO_2_ responses that were on average more negative than responses elicited in vehicle-injected animals. Mean ± SE; * *p* < 0.05; ** *p* < 0.01; one-way repeated-measure ANOVA

Prior to injection, there was no significant difference between oxygenation responses in the vehicle- and IL-1β-injected groups (*t* = 2.016, *p* = 0.073). When comparing the changes of the pO_2_ response to stimulation between the two groups with a repeated-measure two-way ANOVA, excluding the normalised pre-injection time point, there was a significant effect of IL-1β injection on the pO_2_ response (*F*(1) = 6.068, *p* = 0.039), although there was no effect of time (*F*(5) = 0.9227, *p* = 0.4764) or any interaction (*F*(5) = 0.5207, *p* = 0.759) (Fig. 7). When separately analysed for changes within each of the groups as a result of treatment, there was no significant reduction in the size of the pO_2_ response to whisker stimulation (within-group analyses; *F* = 2.488, *p* = 0.0515), but the injection of IL-1β led to a marked reduction in the pO_2_ response over time (*F* = 3.562, *p* = 0.012) (Fig. 7). This reduction was significant after 3 h (Bonferroni multiple comparisons).

### Striatal injection of IL-1β does not significantly affect stimulus-evoked MUA in the cortex

**Fig. 8:**
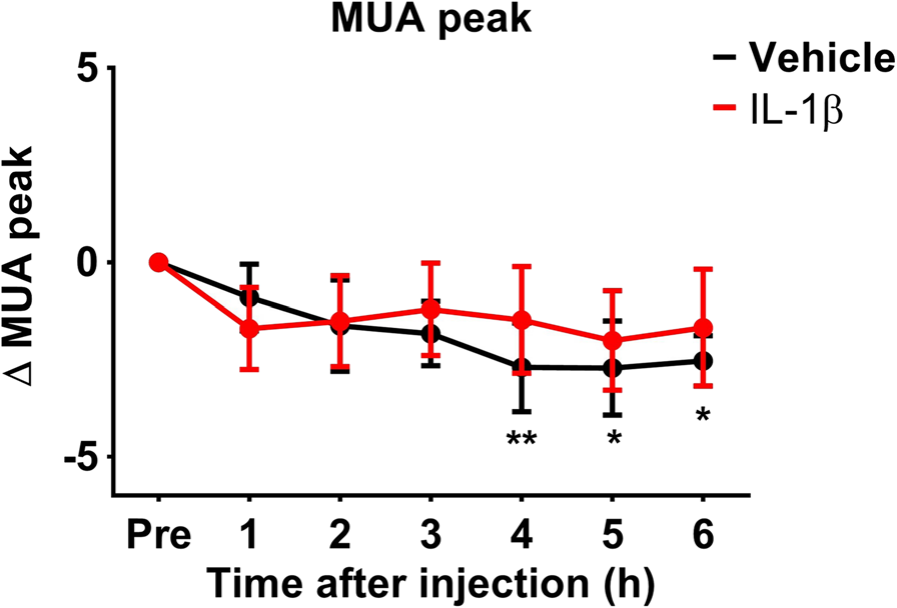
Stimulus-evoked peaks of MUA activity. The frequency of spikes at stimulus onset decreased over time in both IL-1β- and vehicle-injected animals. Mean ± SE; * *p* < 0.05; ** *p* < 0.01; one-way repeated-measure ANOVA

Following the finding that stimulus-evoked gamma- and beta-frequency band activity (which are indicative of neural inputs to the cortex) as well as the haemodynamic response were reduced over time after injection of IL-1β, we investigated whether the level of MUA, which reflects the function of the cell bodies rather than afferent input, was affected. The frequency of spikes at stimulus onset decreased over time in both IL-1β- and vehicle-injected animals with a significant effect of time (*F*(6) = 3.73, *p* = 0.004) (Fig. 8); however, there was no statistically significant difference between the two groups’ gradual decline (*F*(1) = 0.07098, *p* = 0.797).

Interestingly, in the within-group analysis, only the vehicle group showed a significant decrease in MUA; this reduction occurred between 4 and 6 h (repeated-measure one-way ANOVA comparing the pre-injection trials with subsequent measures of the MUA peak after the injection; *F*(*r*) = 4.793, *p* < 0.01; 4 h, *t*(*r*) = 4.091; 5 h, *t*(*r*) = 4.123; 6 h, *t*(*r*) = 3.841; all *p* < 0.05). By contrast, IL-1β did not induce a significant reduction in stimulus-onset spike rate. These data suggest that the capacity of neurons in the cortex of IL-1β-injected animals to produce MUA did not significantly decrease over time, although there was an effect in vehicle-treated animals.

### Striatal injection of IL-1β leads to a decoupling of LFP oscillations and pO_2_ responses

IL-1β-injected animals exhibited markedly lower stimulus-evoked pO_2_ responses and gamma and beta activity at approximately the same time point (3 h), so we investigated whether the size of pO_2_ responses and the size of their corresponding gamma or beta activity peaks were correlated. Previous reports have noted a correlation between stimulus-evoked gamma (and to a lesser extent, beta) activity peaks and the corresponding response in perfusion in healthy animals [15].

**Fig. 9:**
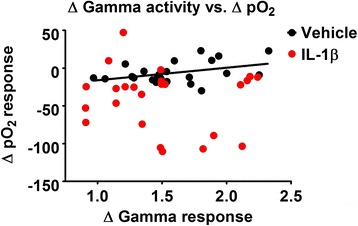
Gamma-frequency band activity coupling with pO_2_ responses. Each data point represents an hourly averaged response from animals injected with either vehicle (*black*) or IL-1β (*red*), normalised to the animal’s pre-injection response. Coupling between gamma neural activity and the corresponding pO_2_ response is maintained in vehicle-treated animals (*black line* is line of best fit), whereas there is no correlative relationship seen between these parameters in IL-1β-treated rats. *n* = 4 animals for each group; six responses per animal (one per hour post-injection)

In vehicle-treated animals, the size of the pO_2_ responses correlated with the sizes of corresponding gamma peaks (*r* = 0.4070, *p* = 0.048) (Fig. 9). However, in IL-1β-injected animals, there was no such correlation between gamma oscillations and the corresponding pO_2_ response (*r* = −0.1192, *p* = 0.579), indicating that striatal IL-1β interfered with the normally tightly coupled relationship between neural activity and the vascular responses.

## Discussion

In this study, we investigated how direct injection of IL-1β in the brain affects the haemodynamic response, neuronal activity and tissue oxygen perfusion. To do this, we recorded haemodynamic responses to whisker stimulation in rats receiving intra-striatal injections of vehicle or IL-1β. Injection of IL-1β led to a decline in the haemodynamic response within hours of injection, and the extent of this decline correlated with levels of neuroinflammation, characterised by increases in neutrophil infiltration and BBB breakdown in the ipsilateral cortex. Further assessment of cortical function using a Clark-style pO_2_ probe (APOX), which simultaneously recorded electrophysiological activity and local tissue oxygenation, suggested that gamma- and beta-band LFP activity may be disrupted by the striatal injection of IL-1β. pO_2_ recordings showed that local tissue oxygenation responses declined within a few hours of IL-1β injection in parallel with the drop in LFP activity. Interestingly, MUA did not seem to be affected by the injection of IL-1β.

**Fig. 10:**
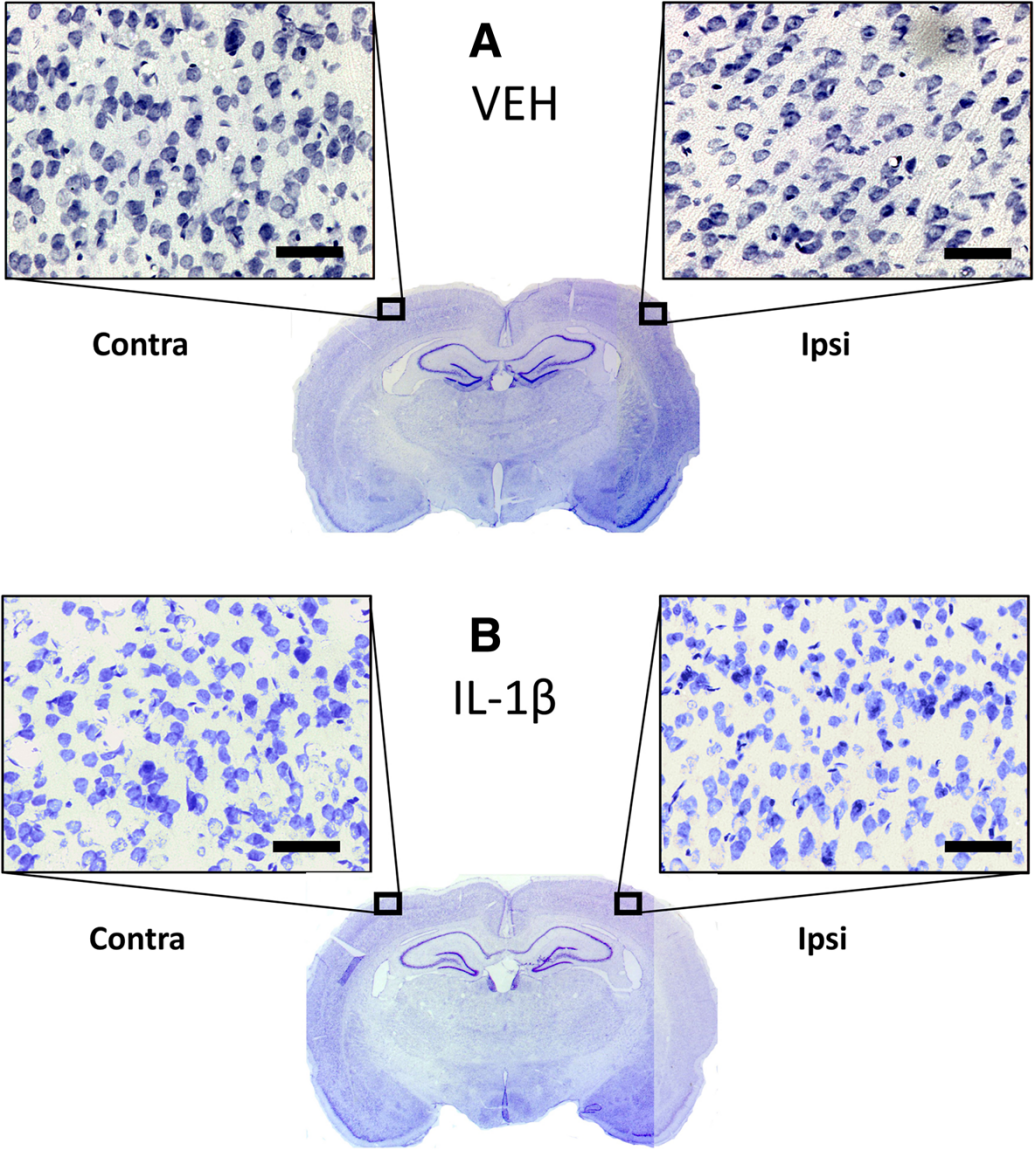
Cresyl violet-stained sections to assess cell death. Both coronal rat brain sections are taken at the level of the barrel cortex where imaging or APOX electrode recordings have taken place. **a** shows an example from the vehicle group and **b** from the IL-1β-treated group. Ipsilateral is the side where the injection and data acquisition took place. Neither hemisphere of both groups showed signs of cell death 6 h after injection. *Scale bar* = 50 μm

No overt cell death (assessed by propidium iodide, Flouro-Jade C and cresyl violet staining) was observed 6 h after injection in any of the brains studied, whether receiving vehicle or IL-1β injections (Fig. 10). The absence of cell death in response to direct injection of IL-1β into the brain supports previous data [2–4], and the BBB breakdown and neutrophil infiltration observed are similar to those reported previously [9, 10]. Inflammatory responses induced by intracerebral IL-1β administration peak between 4 and 8 h after injection and subsequently subside and fully resolve by ~24 h. Whether the acute effects of IL-1β on cortical perfusion and/or neurovascular coupling seen in this study persist beyond 6 h is not known, and such a study would require separate cohorts of animals to those in which acute changes are observed since it would be impossible to maintain animals for extended periods under anaesthesia. However, one would imagine that persistent reductions in perfusion beyond 6 h would be expected to result in some degree of neuronal loss, and as discussed above, previous studies show that direct injection of IL-1β into the brain does not result in any cell death, even when assessed at 24 h after injection.

Although correlation does not prove causation, our data strongly suggest that IL-1β has a negative effect on the haemodynamic response. The mechanisms through which IL-1β could impair haemodynamic coupling remain unclear, although effects on the cerebrovasculature mediated via release of prostaglandins and nitric oxide [25, 26] could be involved. Alternatively, febrile effects of IL-1β (resulting in increased heart rate and core temperature) could have indirect cerebrovascular actions that affect cerebral haemodynamics. It is also possible that IL-1β directly affects neuronal transmission. In the somatosensory cortex, stimulus-evoked gamma-band LFP activity is particularly associated with the rhythmic firing of γ-aminobutyric acid (GABA)ergic interneurons [27, 28]. *N*-Methyl-d-aspartate (NMDA) glutamate receptor-mediated currents have been reported to reduce the power of gamma-band activity [29], whereas the administration of NMDA antagonists increase cortical gamma activity [30–32]. IL-1β potentiates cortical NMDA channels via a Src-ceramide-dependent upregulation of tyrosine kinase activity [33]. Thus, IL-1β could impact on neural oscillations such as gamma-band oscillatory activity via changes in NMDA activity and, given the usually tightly associated relationship between gamma activity and cortical haemodynamics [18], interfere with neurovascular coupling. Recent data also report the effects of IL-1 on pericyte function [34]. Given the increasing recognition of a role for pericytes in maintaining cerebral blood flow and BBB integrity [35], it is intriguing to speculate that the actions of IL-1 seen here are mediated, at least in part, through a direct effect on pericyte function.

Although the direct injection of IL-1β does not result in any overt cell death itself, our findings have important implications for neurological conditions where raised levels of IL-1 have been reported, including stroke and epilepsy. Related to this, we demonstrated some time ago that co-administration of IL-1β with the glutamate agonist AMPA results in an exacerbation of neuronal cell death [3]. Subsequently, we showed that the increased cell death was due to an exacerbation in seizure activity [36]. However, mechanisms by which the seizures result in neuronal loss are not known. Based on our observations here, one hypothesis might be that there is a lack of adequate perfusion to meet the increased energy demand resulting from the seizures. This would result in neurovascular uncoupling, an increase in glutamate and enhanced activation of NMDA receptors, resulting in excitotoxicity and neuronal death. This hypothesis is supported by the finding that block of NMDA receptors prevents the AMPA+IL-1-induced cell death [2].

From this study and many others, it is clear that IL-1 plays a key role in the brain, yet the exact target cells remain elusive. Precise localisation of the signalling receptor (IL-1R1) for IL-1 has proven challenging, in part because very few receptors are required to produce a biological response; hence, expression levels can be extremely low. In this study, the IL-1 could be acting on one or more cells within the neurovascular unit, and future studies using cell-specific receptor-deficient mice [37] will enable this to be resolved.

In summary, although the mechanisms behind the effect of IL-1β on cortical perfusion and neural activity remain to be determined, our findings have important implications for the many neurological disorders in which IL-1β is known to have a key role. More specifically, timely interventions to block IL-1, for example, with IL-1 receptor antagonist (IL-1Ra), may lead to improved perfusion and the maintenance of neurovascular coupling, preventing cell death and loss of function.

## Conclusions

In this study, we show that striatal injection of the pro-inflammatory cytokine IL-1β has deleterious effects on LFP activity and haemodynamic responses within the ipsilateral cortex. Moreover, as MUA (which is representative of neuronal function) remains unaffected by IL-1β at 6 h after injection, we conclude that although the neurons in the cortex of IL-1β-injected animals are still intact and functional, the neurovascular coupling between LFP activity and the corresponding influx of oxygenated blood is acutely lost. These findings may have implications for diseases in which acute neuroinflammation takes place—especially disorders such as stroke, wherein cerebral blood flow is already compromised.

## Abbreviations

ANOVA: Analysis of variance
APOX: Action potential-oxygen
BBB: Blood-brain barrier
BOLD: Blood-oxygen-level-dependent
BSA: Bovine serum albumin
DAB: Diaminobenzidine
dH_2_O: Distilled water
ECG: Electrocardiogram
EEG: Electroencephalogram
GABA: γ-Aminobutyric acid
HbO_2_: Oxyhaemoglobin
HbT: Total haemoglobin
H_2_O: Water
IgG: Immunoglobulin G
IL-1β: Interleukin-1β
IL-1Ra: Interleukin-1 receptor antagonist
LFP: Local field potential
MUA: Multi-unit activity
NMDA: *N*-Methyl-d-aspartate
OIS: Optical imaging spectroscopy
PBS: Phosphate buffer solution
pO_2_: Partial pressure oxygen
ROI: Region of interest
SD: Standard deviation
SpO_2_: Peripheral capillary oxygen saturation
